# Acceptability of smokers of a conceptual cigarette tracker as wearable for smoking reduction

**DOI:** 10.1186/s13104-022-05935-2

**Published:** 2022-02-10

**Authors:** Jumana Antoun, Rana Shehab, Georges Sakr, Sani Hlais, Mariette Awad, Maya Romani

**Affiliations:** 1 Department of Family Medicine, Faculty of Medicine, American University of Beirut, Beirut, Lebanon; 2 Faculty of Engineering, Saint-Joseph University of Beirut, Beirut, Lebanon; 3 Faculty of Medicine, Saint-Joseph University of Beirut, Beirut, Lebanon; 4 Maroun Semaan Faculty of Engineering and Architecture, American University of Beirut, Beirut, Lebanon

**Keywords:** Smoking cessation, Smoking reduction, Wearable, Behavior change, TAM model

## Abstract

**Objective:**

The study aims to explore smokers' acceptance of using a conceptual cigarette tracker like a cigarette filter for smoking cessation using the Technology Acceptance Model (TAM). Smokers presenting to the family medicine clinics at a tertiary care center were asked to complete an anonymous questionnaire.

**Results:**

A total of 45 participants were included. Two-thirds of the smokers reported that they would like to try such a tracker and perceived its usefulness in reducing the number of daily cigarettes consumed and increasing the motivation to join a smoking cessation program. A range of 40–50% of the participants had a neutral attitude towards the visibility of the tracker and its effect on social acceptance and self-image. The structural equation model with latent variables path analysis showed that only perceived usefulness correlated to the intention to adopt with statistical significance. Visibility was correlated with intention to adopt with a marginal p-value of 0.061. Driven by perceived usefulness, smokers may buy or try a cigarette tracker for smoking reduction or cessation.

**Supplementary Information:**

The online version contains supplementary material available at 10.1186/s13104-022-05935-2.

## Introduction

Smoking is a public health concern worldwide. Smoking cessation is challenging for the smoker on many levels, including the physical and psychological aspects. Interventions that include both behavioral and pharmacologic therapies show success rates. Despite all the behavioral and pharmacological interventions, quit rates are maximum at 40%. Therefore, there is still room to develop new interventions. Mobile applications have shown promising results with a range of 13–24% quit rate [1, 2]. Using checklists and recording the number of puffs that one has consumed and coping mechanisms to resist cravings are the most frequently utilized elements in cigarette smoking cessation apps [1, 3–5]. Even though these mechanisms are efficient, relying on user self-reporting cigarette intake appears burdensome.

Alternative ways to promote automated self-monitoring are needed to reduce users’ burden of inputting behavioral data. Wearables are possible tools that could support the automated self-monitoring of smoking. Wearable trackers have been used to help consumers follow a healthy lifestyle, increase their physical activity [6] and decrease in weight [7], especially when combined with a smartphone application [8, 9]. In 2019, a systematic review summarized the various attempts to use wearable sensors to detect a smoking episode, such as the use of a lighter, wrist sensors based on hand to mouth proximity, respiratory signals based on a belt sensor, acoustic signals based on throat sensors, and others [10].

Our research team is working on a new prototype of a cigarette tracker based on heat and pressure sensors. The cigarette tracker would resemble a cigarette filter, a small plastic piece that holds the cigarette to reduce the amount of tar smokers inhale. The tracker will be linked to a smartphone application that follows behavioral change theories. The study explores smokers' acceptance of using a conceptual cigarette tracker like a cigarette filter for smoking cessation using the Technology Acceptance Model (TAM).

## Main text

### Methods

This study is a cross-sectional anonymous survey-based among smokers presenting to the family medicine clinics at the American University of Beirut in Lebanon. All patients were approached to participate in the research at the triage station if they were current smokers (cigarette, Hubble bubble, or electronic cigarettes). Inclusion criteria included adults aged 18 and above who are current smokers. Illiterate patients were excluded as they needed to read and fill the questionnaire independently. The nurse introduced the research, explained as needed, and asked if they would like to participate. If they agree, they were provided with the informed consent and questionnaire. The patients were asked to fill it privately and drop it in a closed box.

The Institution Review Board granted ethical approval at the American University of Beirut.

### Questionnaire

The questionnaire (Additional file 1) included three sections: (1) a visual of the potential cigarette tracker prototype and a description of its use, (2) demographics including gender, age, level of education, monthly income, number of daily cigarette consumption, ever use of wearables and accepted cost of such a tracker, and (3) questions related to the acceptance of the tracker using the TAM model framework.

TAM is a commonly used model to explain users' acceptance of new technology in healthcare [11]. According to TAM, perceived ease of use and perceived usefulness will determine the attitude and intention to use, whether the consumer will use the technology [12]. Perceived usefulness is described as “the prospective user’s subjective probability that using a specific application system will increase his or her performance” [12]. Perceived ease of use is defined as “the degree to which a person believes that using a particular system would be free of effort” [12]. The visibility factor was added as it is a major human factor that may affect the acceptability of wearables computers [13].

The research team developed the questions and refined using frequent iterative meetings among the research team members (JA, MR, and RS). The research team is an expert in the domain. MR is a smoking cessation specialist. RS is a behavioral counseling smoking cessation nurse in the smoking cessation program. JA is a specialist in health informatics. SH finally reviewed the content from a participant's perspective, and few grammatical and sentence structural changes were done. Participants were asked to answer the questions using a Likert scale from1 to 7. Participants strongly disagreed with the statement if they scored between 1 and 3. A score of 4 was considered a neutral position. A score of 5–7 was considered a strong agreement with the statement.

### Statistical analysis and sample size calculation

Descriptive data of the demographics and the various acceptance model questions were performed with frequencies for categorical variables and means for continuous variables. Linear regression analysis and structural equational modeling (SEM) were used to test the reliability and validity of the framework using AMOS. For SEM, the suggested minimum for sample size ranges from 3 to 20 times the number of variables. The model has 18 variables, and considering a ratio of 3:1, we need a sample size of 54. SPSS version 23.0 was used for descriptive statistics and exploratory factor analysis, and AMOS version 21.0 was used for SEM. Statistical significance was set at p < 0.05.

### Results

A total of 45 smokers were included. Table 1 shows the demographics. The mean age was 36.1, with a standard deviation of 13.7 years. The majority (81.4%) achieved a college or post-graduate degree. \Two-thirds of the participants (65.7%) had an income above 1000$ (65.7%). The minimum wage is 450 dollars in Lebanon at the time of conduction of the study [14]. The mean cigarette intake was 18.1 cigarettes per day (SD 17.2).

**Table 1:** Demographics of the participants

	Total N	N	Percent
Gender	36		
Female		15	33.3
Male		21	46.7
Education	43		
High school and less		5	11.1
Technical		3	6.7
College		22	48.9
Postgraduate		13	28.9
Monthly Income	38		
< 500$		8	17.8
500–999$		5	11.1
1000–2000$		14	31.1
> 2000$		11	24.4
		**Mean**	**SD**
Age	44	36.1	13.7
Number of daily cigarette consumption	41	18.1	17.2

Table 2 shows the participants’ responses to the various questions related to the acceptance of the cigarette tracker based on the TAM model. Participants would accept the tracker's price to be between 20 and 50$. Interestingly, 65% said they would like to try such a tracker. Two-thirds of the participants perceived the usefulness of the tracker to reduce the consumed daily cigarettes and increase the motivation to join a smoking cessation program. Only half of the participants agreed with the perceived ease of use of the tracker. Very few (11.9–13.6%) had negative attitudes towards using the technology. More people (65.1%) were likely to try the tracker than definitely buy the tracker (50.0%). A range of 40–50% of the participants had a neutral attitude towards the tracker's visibility and its effect on social acceptance and self-image.

**Table 2:** Participants responses to the acceptance of the cigarette tracker based on the TAM model

	Agree (%)	Neutral (%)	Disagree (%)
Perceived usefulness			
A cigarette tracker could help me reduce the number of cigarettes consumed per day	60	20	20
A cigarette tracker could help me stop smoking	40	28.9	31.1
A cigarette tracker could increase my motivation to join a smoking cessation program	64.4	22.2	13.3
A cigarette tracker can help me track my smoking habits	55.6	11.1	33.3
A cigarette tracker can help me improve my health	57.8	15.6	26.7
Perceive ease of use			
Using the tracker is simple	51.2	32.6	16.3
Using the tracker is self-explanatory	51.2	23.3	25.6
It is easy to carry the tracker	54.5	20.5	25.0
It is comfortable to use the cigarette tracker	48.8	34.1	17.1
Attitudes towards technology			
I like the idea of using a cigarette tracker	47.6	40.5	11.9
Overall, I have positive attitude towards the use of a cigarette tracker	52.3	34.1	13.6
Intention to adopt			
I would most probably buy a cigarette tracker	45.2	35.7	19.0
I would definitely buy a cigarette tracker	50.0	28.6	21.4
I would like to try a cigarette tracker	65.1	11.6	23.3
Visibility of the tracker			
Cigarette trackers may be socially unacceptable	23.3	39.5	37.2
Cigarette trackers are visible to others	45.2	28.6	26.2
The appearance is aesthetically appealing to me	22.7	52.3	25.0
The use of the tracker will improve my self-image	31.1	44.4	24.4

Only 6 participants (13.3%) owned a wearable: Apple watch (1), Fitbit (4) or Polar (1). The participants were split equally when asked whether a cost of 100$ for the tracker would hinder them from buying the tracker. Women (62.5%) were more likely to report that 100$ may prevent them from buying the tracker than men (37.5%), *X*^2^(1, N = 33) = 6.945, *p* = 0.013. When asked about the accepted cost of the tracker, participants proposed a range of 20–50$ as an acceptable price of the tracker.

A structural equation model with latent variables path analysis was performed to predict adoption or intention (INT). The latent variables are perceived usefulness (PU), perceived ease of use (PEU), attitude (A), and visibility (V). Intention to adopt was computed as the sum of the three intention questions. Question V2 was put in the model; however, its p-value > 0.05 and was removed from the final model. The measurements of goodness of fit were as follows: X2(df = 32) = 43,299, p = 0.088; RMSEA = 0.102, CFI = 0.940. Only CFI, which is not very sensitive to sample size, showed goodness of fit.

The hypothesized model is given by:

$$ PU = \sim {\text{PU}}1{ } + {\text{ PU}}2{ } + {\text{ PU}}3{ } + {\text{ PU}}4{ } + {\text{ PU}}5 $$

$$ PEU = \sim {\text{PEU}}1{ } + {\text{ PEU}}2{ } + {\text{ PEU}}3{ } + {\text{ PEU}}4 $$

$$ A = \sim A1 + A2 $$

$$ V = \sim V1 + V3 + V4 $$

$$ INT\sim PU + PEU + A + V $$

**Fig. 1:**
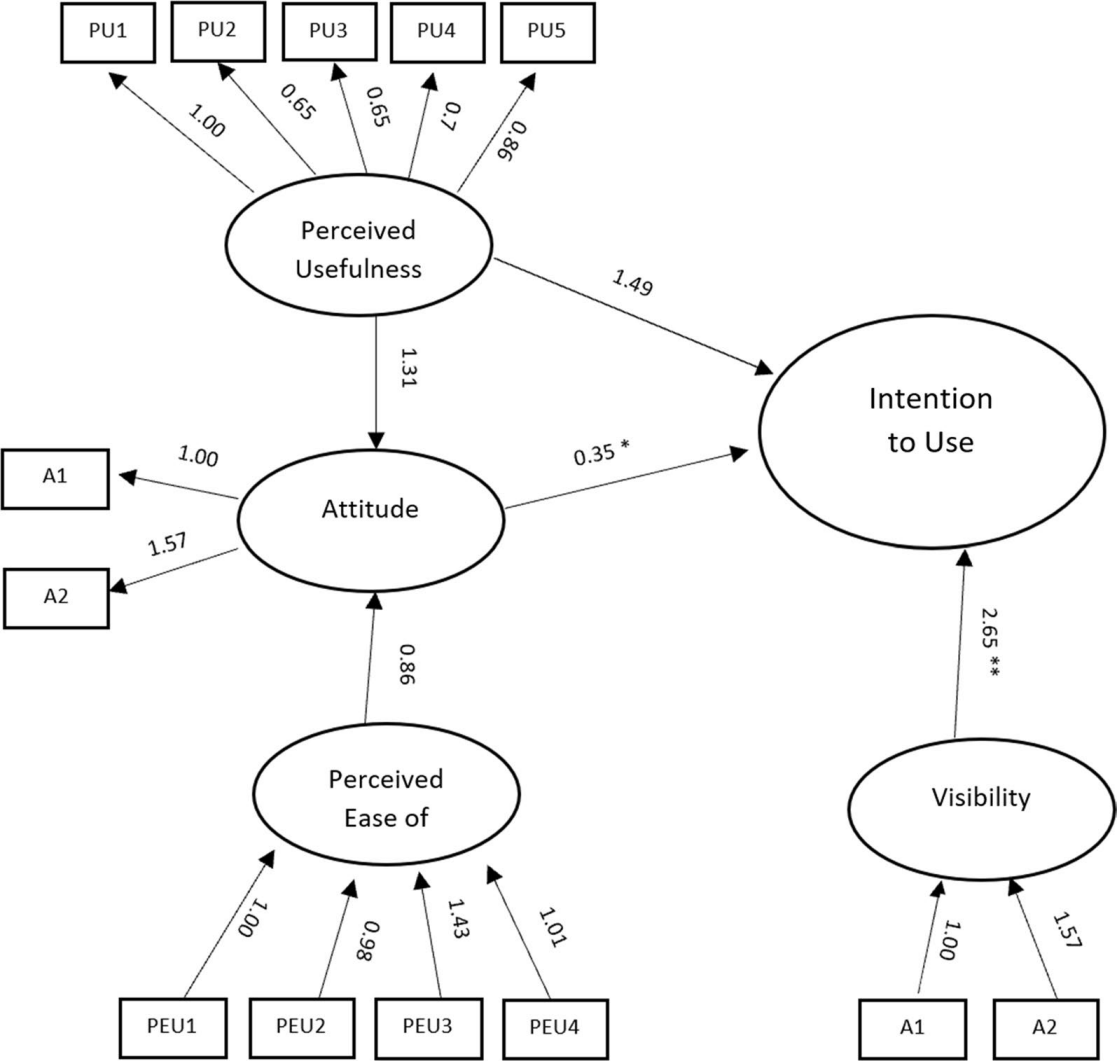
SEM model showing the various regression coefficients among the various variables. All regression coefficients are significant at p < .05 except *p-value = 0.7 and **p-value = 0.06

Figure 1 shows the SEM model with the various regression coefficients. Only perceived usefulness correlated with intention to adopt with statistical significance. Visibility was correlated with intention to adopt with a p-value of 0.061. The SEM model proved the TAM model relationships except for the non-significant relationship between the attitude and intention of use.

### Discussion

Smoking cessation is challenging. This cross-sectional survey-based study aimed to measure smokers' acceptance to the use of a conceptual cigarette tracker in the form of a cigarette filter for smoking cessation/reduction using the TAM model as a framework. Two-thirds of the smokers would try the tracker. Smokers had a positive attitude towards the tracker and its perceived usefulness. They were less positive about its ease of use and neutral about the visibility of the tracker and its effect on social acceptance and self-image. Perceived usefulness was the most important predictor of the use of the tracker.

The TAM model has been used frequently in the adoption of technology in healthcare [11] and for smoking cessation [15]. Similar to our study, perceived usefulness was also an important predictor of the use of a potential SMS-assisted smoking cessation program [15]. In a study in China and Pakistan, both perceived ease of use and perceived usefulness positively impacted users' intention to use mHealth technologies for smoking cessation [16]. Regarding the use of wearables, similar to our study results, a study among 927 people who purchased their smartwatch or smartband in South Korea has also shown that perceived usefulness was the most influential predictor of attitude and intention [17]. A recent meta-analysis among users’ acceptance of consumer-oriented health information technologies based on TAM has shown that perceived usefulness has a stronger relationship with attitude and behavioral intention than perceived ease of use [18]. Characteristics of the technology, the context, the user, perceived benefits and risks, and social factors may influence the adoption of health and fitness wearables [19, 20].

Visibility of the technology and social factors such as social norms and image regulation were lumped into one component in this study analysis. This component was correlated with intention to use yet with a p-value of 0.06. The social aspect of new technologies has been tested among smartwatches, smart glasses, smart clothing, and health and fitness wearable devices [21]. For example, both perceived usefulness (β = 0.113) and visibility (β = 0.248) showed a positive effect on the intention to use smartwatches; nevertheless, only the impact of visibility reached statistical significance [22]. The effect of visibility on technology adoption may be related to the context and type of technology. While the look-and-feel of a smart glass was the most frequently mentioned factor for adoption, other factors beyond visibility were mentioned for smartwatch adoption, such as the availability of fitness applications [23]. Furthermore, the visibility and social aspect of technology may be related to society's familiarity with the technology. The cigarette filter is not a new technology used among smokers [24]. This could have also contributed to the lack of ease of use on the adoption of the cigarette tracker. Only few (less than 25%) participants had concerns that the tracker may not be simple, self-explanatory, easy, or comfortable to carry.

### Future implications

From a theoretical point of view, this cigarette tracker would be innovative and add to the list of behavioral interventions intended for smoking cessation. It is scalable and can address many smokers who are still reluctant to set a quit date. Furthermore, this study shed light on marketing e-health programs or wearables where the consumer is more likely to use the technology if they perceive its usefulness. Finally, qualitative studies could better understand the perspective of those who do not intend to use the cigarette trackers.

### Conclusion

The use of a conceptual cigarette tracker for smoking cessation may be acceptable by smokers. Smokers are interested in the usefulness and benefits of the tracker. Visibility and social acceptance of the tracker may play a lesser role in their adoption.

## Limitations

This study asked the participants about their adoption of a conceptual wearable. They were given a description of the wearable and a picture of how it will look. This could have affected their responses and explained why the study could not establish statistical significance among the various factors of the TAM model. Furthermore, it was conducted at a single institution in Beirut and may not generalize to the general population and different cultures.

## Supplementary Information

**Additional file 1.** Appendix 1: Questionnaire.

## Data Availability

The datasets used and/or analysed during the current study available from the corresponding author on reasonable request.

## Abbreviation

TAM: Technology Acceptance Model
