# Supplementary material for: Acceptability of smokers of a conceptual cigarette tracker as wearable for smoking reduction
Source: BMC Res Notes. 2022 Feb 10;15:38. doi: 10.1186/s13104-022-05935-2 (PMC8832834; doi:10.1186/s13104-022-05935-2)
Supplement: Supplementary file 1 — Additional file 1. Appendix 1: Questionnaire. [file 13104_2022_5935_MOESM1_ESM.docx]

Appendix 1: The Questionnaire No: ______

Cigarette tracker is a potential innovative technology product that can be used to track the number of cigarettes consumed during the day. It will be associated with an application that tracks your daily consumption of cigarettes, gives you individualized feedback and motivation, helps you in setting goals and connects you to other tracker users. It will most probably look like a cigarette filter as shown in the below figure. You buy the tracker once for a certain amount of money. It is charged as needed.


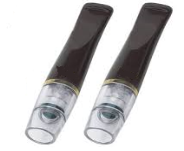

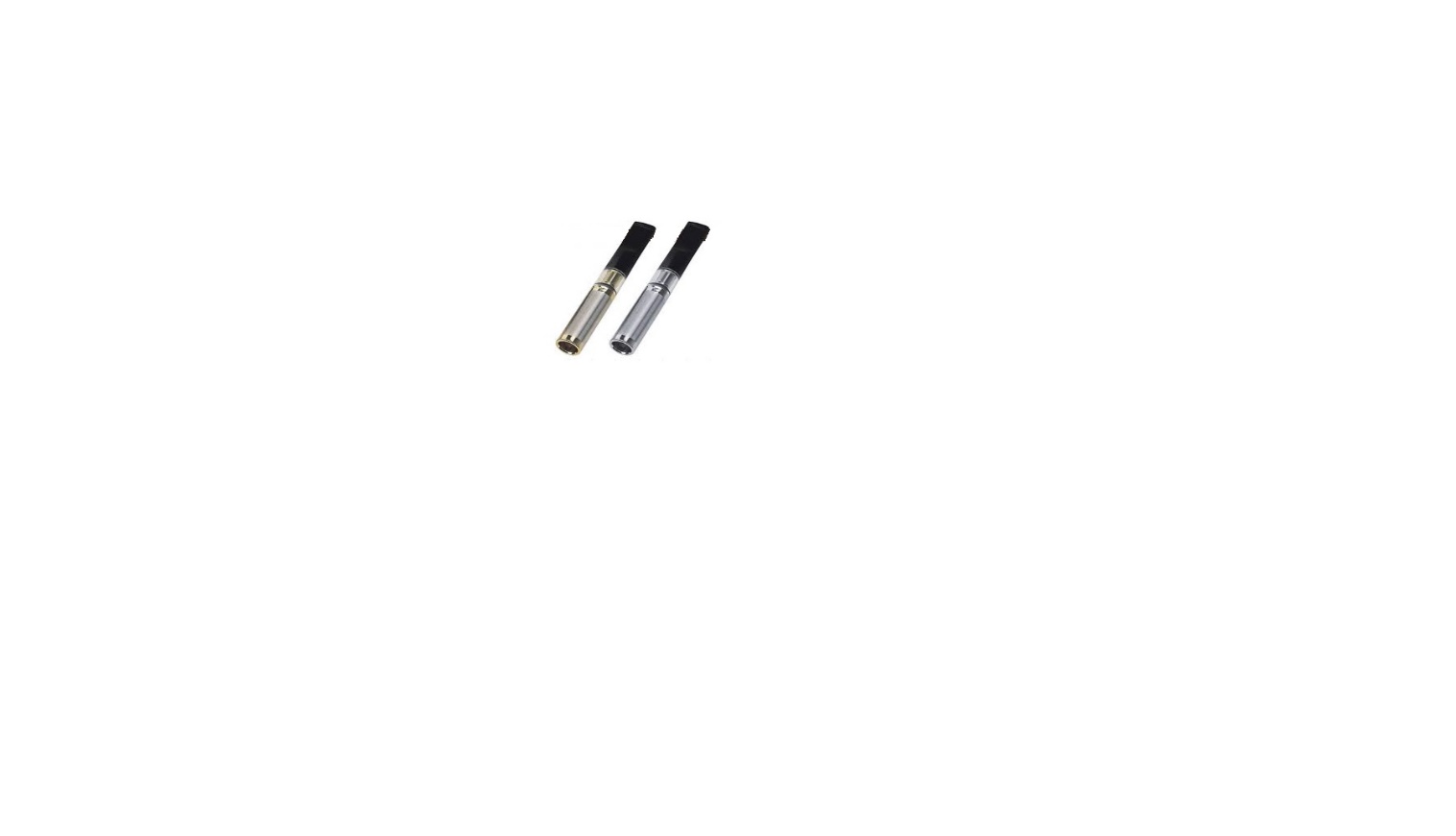


Please indicate your degree of agreement or disagreement with the statements shown below

|  | 1 | 2 | 3 | 4 | 5 | 6 | 7 |
| --- | --- | --- | --- | --- | --- | --- | --- |
|  | Strongly disagree |  |  | Neutral |  |  | Strongly agree |
| A cigarette tracker could help me reduce the number of cigarettes consumed per day. |  |  |  |  |  |  |  |
| A cigarette tracker could help me stop smoking. |  |  |  |  |  |  |  |
| A cigarette tracker could increase my motivation to join a smoking cessation program. |  |  |  |  |  |  |  |
| A cigarette tracker can help me track my smoking habits. |  |  |  |  |  |  |  |
| A cigarette tracker can help me improve my health |  |  |  |  |  |  |  |
| Using the tracker is simple. |  |  |  |  |  |  |  |
| Using the tracker is self-explanatory. |  |  |  |  |  |  |  |
| It is easy to carry the tracker. |  |  |  |  |  |  |  |
| It is comfortable to use the cigarette tracker. |  |  |  |  |  |  |  |
| I like the idea of using a cigarette tracker. |  |  |  |  |  |  |  |
| Overall, I have positive attitude towards the use of a cigarette tracker. |  |  |  |  |  |  |  |
| I would most probably buy a cigarette tracker |  |  |  |  |  |  |  |
| I would definitely buy a cigarette tracker |  |  |  |  |  |  |  |
| I would like to try a cigarette tracker |  |  |  |  |  |  |  |
| Cigarette trackers may be socially unacceptable. |  |  |  |  |  |  |  |
| Cigarette trackers are visible to others. |  |  |  |  |  |  |  |
| The appearance is aesthetically appealing to me. |  |  |  |  |  |  |  |
| The use of the tracker will improve my self-image. |  |  |  |  |  |  |  |

Please answer the following questions:

**Gender:** 1. Female 2. Male

**Age:** ________

**Level of education:** 1. High school or less 2. University or college 3. Post graduate degrees

4. Technical training

**Monthly income:** 1. <500$ 2. 500-999$ 3. 1000-2000 4 4. >2000$

**Number of cigarettes per day: ­­­­­**_______

**Do you own any type of tracker or wearable such as Fitbit, Jawbone, Apple watch?** 1. No 2. Yes.

If yes, please name it ____________

**If the cost of the tracker is 100$, would this cost hinder you from buying the tracker?**  1. No 2. Yes

If yes, what is the accepted cost for you to buy the tracker? ---------$
